# The Elements of Bacteriological Technique

**Published:** 1904-03

**Authors:** 


**The Elements of Bacteriological Technique.** By J. W. H. Eyre, M.D. Pp. 371. Philadelphia and London: W. B. Saunders and Co. 1902.

This book is intended to be a "laboratory guide for the medical, dental, and technical student." It is founded upon the author's notes for his own classes of practical bacteriology. It is a thoroughly practical book, every apparatus and manipulation being fully and carefully described. The illustrations are very numerous, and are simple, clear, and to the point, and will be found of great assistance to those who wish to make their own apparatus. As a reference book for methods and media it is excellent, and the information is well classified and easily found when required. It is not intended to be a reference book for running down and identifying any particular organism, but the methods of studying the properties of an organism that are being investigated are excellent. This book should find a place on the shelves of every clinical laboratory.

